# Differential Bone Marrow Homing Capacity of VLA-4 and CD38 High Expressing Chronic Lymphocytic Leukemia Cells

**DOI:** 10.1371/journal.pone.0023758

**Published:** 2011-08-18

**Authors:** Gabriele Brachtl, Karine Sahakyan, Ursula Denk, Tamara Girbl, Beate Alinger, Sebastian W. Hofbauer, Daniel Neureiter, Josefina Piñón Hofbauer, Alexander Egle, Richard Greil, Tanja Nicole Hartmann

**Affiliations:** 1 Laboratory for Immunological and Molecular Cancer Research, Third Medical Department with Hematology, Oncology, Hemostaseology, Infectiology and Rheumatology, Private Medical University Hospital, Salzburg, Austria; 2 Institute of Pathology, Paracelsus Medical University, Salzburg, Austria; Emory University, United States of America

## Abstract

**Background:**

VLA-4 and CD38 predict a poor clinical outcome in chronic lymphocytic leukemia (CLL). We used CLL samples with discordant VLA-4/CD38 risk to address their individual roles in human bone marrow infiltration (BM), CLL cell homing to murine BM, and in supportive CLL cell-stromal cell interactions.

**Methods:**

VLA-4, CD38, and Ki-67 expression was measured in CLL cells from peripheral blood (PB) and bone marrow (BM) aspirates. CLL BM infiltration rates, routinely determined by Pathology, were correlated to VLA-4 and CD38 expression. Short-term homing capacity of CLL cells was evaluated by adoptive transfer experiments. CLL cell viability and adhesion in stromal cell co-culture was determined.

**Results:**

About 20% of CLL samples in our cohort displayed discordant VLA-4 and CD38 risk, with either high VLA-4 and low CD38 risk or vice versa. Using particularly such samples, we observed that VLA-4, and not CD38, was responsible for recirculation of CLL cells to murine BM. Human BM infiltration was also significantly higher in patients with high VLA-4 risk but not high CD38 risk. However, both molecules acted as independent prognostic markers. While both VLA-4 and CD38 expression were increased in BM-derived CLL cells, and VLA-4+ and CD38+ subpopulations showed enriched Ki-67 expression, VLA-4 did not contribute to CLL cell protection by stromal cells in vitro.

**Conclusions:**

Our data argue for a prominent role of VLA-4 but not CD38 expression in the homing of CLL cells to BM niches and in human BM infiltration,but only a limited role in their protection by stromal cells.

## Introduction

Chronic lymphocytic leukemia (CLL) is characterized by the accumulation of CD5+ B lymphocytes in the blood, bone marrow (BM) and secondary lymphoid tissues. According to the current view, CLL cells are highly dependent on microenvironmental interactions that provide proliferative and prosurvival stimuli to the malignant cells. CLL is a heterogeneous disease with a highly variable clinical course and a number of molecular prognostic markers have been identified to help determine that course. Among these are VLA-4 and CD38,[1]–[3] two surface molecules that are believed not only to be mere markers of disease aggressiveness but also to play a role in CLL pathogenesis. VLA-4 (α4β1, CD49d/CD29) is the exclusive member of the α4 integrin subfamily expressed by CLL cells and has recently been identified as a negative prognostic marker in this disease[2], [3]. VLA-4 plays a key role in the retention of hematopoietic progenitors in BM stroma, which is required for normal early B cell development.[4] The second prognostic indicator, CD38, is a promiscuous glycoprotein that functions as an ectoenzyme, a surface receptor, and an adhesion molecule.[5] In CLL, CD38 ligation in the presence of IL-2 induces the survival and proliferation of the tumor cells.[6], [7] Furthermore, CLL cells expressing CD38 are thought to have enhanced migratory capacity.[8], [9]

Because VLA-4 and CD38 expression have been described to be highly associated in CLL,[2], [10] we sought to determine their functional consequences in the context of supportive BM niches. For this we employed *in vivo* adoptive transfers of human leukemic cells into immunodeficient mice, *in vitro* flow cytometrical phenotyping of peripheral blood (PB) - and BM-derived CLL cells, and stromal cell co-culture experiments. Moreover, the extent of BM infiltration in CLL patients was correlated to the VLA-4 and CD38 status. Our data suggest that VLA-4 but not CD38 expression plays a prominent role in the homing of high-risk CLL cells to supportive BM niches and in human BM infiltration but only a limited role in supportive CLL cell-stromal cell interactions *in vitro*.

## Methods

### Patient samples

Following written informed consent, samples from 144 CLL patients seen at the University Hospital Salzburg (Table 1 and Table S1, Methods S1) were obtained and cytometrically analyzed for CD38 and VLA-4 (CD49d) expression. Analyzed patients were chemonaive or had received no chemotherapy in the last six months. Peripheral blood mononuclear cells (PBMCs) and mononuclear cells of BM aspirates from CLL patients and PBMCs from healthy donors were isolated by Ficoll density gradient centrifugation and freshly used, or viably frozen in FCS plus 10% DMSO for storage in liquid nitrogen. Thawed cells were cultured in RPMI medium 1640 supplemented with 10% FCS and antibiotics prior to use. Bone marrow biopsies from CLL patients were routinely analyzed by Pathology for CLL infiltration rates.

**Table 1:** Patient characteristics.

	VLA-4 Low/CD38 Low	VLA-4 High/CD38 High	VLA-4 Low/CD38 High	VLA-4 High/CD38 Low	Total
*N = *	83	30	13	18	144
Characteristics	Percent of patients (%)				
*Sex*					
Female	45	43	31	44	43
Male	55	57	69	56	57
*Rai stage*					
0	47	23	46	22	39
I-II	48	53	46	56	50
III-IV	5	23	8	22	11
*Treatment status*					
Chemonaive	70	40	54	39	58
Treated	30	60	46	61	42
Risk parameters					
*IgVH* UMT	10	62	70	54	31
*FISH* High-risk	6	31	27	27	15
*ZAP-70* High-risk	21	75	60	40	38

*IgVH* UMT, *unmutated IgVH; FISH* High-risk, either del17p, del11q or tri12;

### Mice

NOD/NCrCrl- Prkdc (NOD/SCID) mice were purchased from Charles River Laboratories, Germany. Experiments were performed under the approval from the Austrian Animal Ethics Committee. PBMCs (10–25×10^6^) from CLL patients were injected into the tail vein of nonirradiated mice. Cell viability was determined before injection. At indicated time points, animals were sacrificed, cells were recovered from BM, spleen and other murine organs, and human cells were detected by flow cytometry using human-specific antibodies. Cells obtained from mice injected with PBS only were used to exclude false-positive events. Homing rates were normalized to the number of analyzed murine cells and viable injected human cells as described and were comparable to those of previous reports [11]–[13]. In addition, absolute recovery rates of injected human cells in all organs analyzed were calculated using Flow-Count Fluorospheres (Beckman Coulter, FL).

### Immunohistochemistry

Samples from patients (BM) and mice (BM and spleen) were routinely prepared as 5 µm thick paraffin sections. Basic histomorphology was evaluated (hematoxylin and eosin staining). To characterize and quantify the infiltrate of CLL cells, monoclonal antibodies against CD3, CD5, CD10, CD20, CD23 and CD79 (all from Dako, Glostrup, Denmark) and the autostainer system (Dako) was used according the manufacturer's recommendations. VLA-4 expression was detected by polyclonal anti-VLA-4 (AHP1225, Serotec, Duesseldorf, Germany) and CD38 expression by monoclonal anti-CD38 (Eubio). Blind scoring was carried out in the Department of Pathology.

### Ki-67 assessment

For assessment of Ki-67 expression, thawed mononuclear cells from BM aspirates from CLL patients were stained with CD19-PC7, CD5-PC5 and CD38-PE (Beckman Coulter) or VLA-4-PE (or respective isotype control, Becton Dickinson (BD), NJ) before fixation and permeabilization (Cytofix/Cytoperm, BD). Cells were then stained with Ki-67-FITC or control-FITC (BD) before flow cytometrical analysis.

### Cell viability

Cells were stained with Annexin V-FITC (Alexis Biochemicals, Lausen, Switzerland), 7-AAD, anti-CD5-PC7 (Beckman Coulter) and anti-CD19-PE (BD) and analyzed by flow cytometry. Viable cells were defined as Annexin V/7-AAD negative. Where indicated, Flow-Count Fluorospheres were added for calculation of absolute cells numbers. Samples were analyzed using a FC-500 or Gallios flow cytometer and CXP2.2, Gallios Cytometer Software 1.1 (Beckman Coulter) and FlowJo (Tree Star, Ashland, OR).

### Cell culture and Adhesion Assays

For spontaneous apoptosis measurements, freshly isolated PBMCs (10^6^/ml) were cultured in dishes coated with human serum albumin (HSA, Calbiochem, Merck, Darmstadt, Germany). For stromal co-culture experiments, M2-10B4 cells (CRL-1972, ATCC, Manassas, VA) were cultured overnight (300.000 cells per well, 24-well plate) before PBMCs (10^6^/ml) were added. Where indicated, cells were treated with 5 µM fludarabine (Sigma-Aldrich, St. Louis, MO). CLL cells were allowed to adhere to stromal cells for 48 hours before VLA-4 and CD38 expression, viability and adherence were assessed by flow cytometry. To determine the percent of adherent cells, the non-adherent fraction was first separated from the adherent fraction by rigorous washing. The adherent cells were then harvested by trypinization and collected in a separate tube. The number of CLL cells in each fraction was calculated by the use of Flow-Count Fluorospheres. Where indicated, PBMCs were pretreated with 0.3 µg/ml anti-VLA-4 antibodies or isotype control (BD) for 30 minutes prior addition to M2-10B4 cells. To confirm VCAM-1 expression on M2-10B4 cells, stromal cells were harvested using trypsin/EDTA and washed with PBS. Cells were stained with anti-VCAM-1-PE or with respective negative isotype control-PE (both BD) before flow cytometrical analysis

### Ethics

No ethics committee approval is necessary for the experiments of this study using primary human cells and tissues as it is not part of any clinical study. This is confirmed by the ethics committee of Salzburg, Austria (Ref. No. 415-E/1287/8-2011). For experiments with human cells and tissue samples written informed consent was obtained from all patients and healthy donors. The patients agreed that their blood and tissue samples are being used for scientific purposes and they were informed that those samples are only obtained when clinically necessary.

All animal (mouse) experiments were performed under the approval from the Austrian Animal Ethics Committee. The Federal Ministry for Science and Research of Austria granted permission (Ref. No. 14606/9-2005) to perform animal experiments in accordance with Animal Experimentation Law (TVG) (BGBl.Nr.501/1989, last changed to BGBl. I Nr.162/2005).

### Statistical analysis

All statistical analyses were performed using Graphpad Prism (Graphpad Software, La Jolla, CA). All data were tested for normal distribution. Comparisions of normally distributed data from two groups were done with t-tests (Paired or Unpaired t-tests, respectively), while nonparametric data sets were compared using Wilcoxon signed rank test (paired analysis) or Mann Whitney test (unpaired analysis). For comparing three or more groups one-way ANOVA and respective nonparametric tests were used as well as corresponding post hoc tests. Data are presented as scatter, line, or columns plots. Differences were considered statistically significant when P<.05. P-values<.05 are marked with *, <.01 with ** and <.001 with ***.

## Results

### VLA-4 expression is significantly associated with CD38 expression

**Figure 1:**
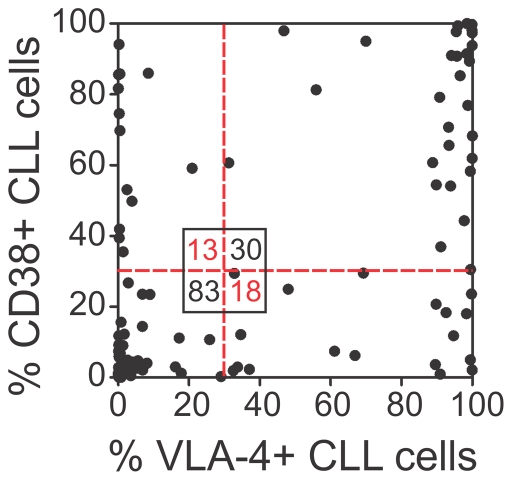
VLA-4 and CD38 expression are associated in CLL cells. Percent VLA-4+ correlated to percent CD38+ CLL cells of 144 CLL patients (Spearman's Rho  =  .6293, P<.0001). Red lines depict cut-offs between low and high-risk samples for each parameter, separating the cohort into four subgroups. Of note, 31 patients in our cohort (21.5%) showed discordant VLA-4 and CD38 risk.

Prior to functional studies, we analyzed the VLA-4 and CD38 expression on CLL cells from the PB of 144 CLL patients. CLL samples with VLA-4 expression on ≥ 30% of the CLL cells were defined as VLA-4 high-risk samples [2], [3]. For definition of CD38 high-risk samples, an equivalent cut-off value of ≥ 30% was used as previously described [1]. Using both parameters as categorical variables, we found a strong association between the VLA-4 and CD38 risk groups (Chi-square Test, Chi-square  =  36.62, p<.0001), and a positive correlation of the individual expression levels (Fig. 1). Nevertheless, 31 patients (21.5%) in our cohort displayed discordant VLA-4 and CD38 expression, with either low VLA-4 and high CD38 risk (VLA-4-/CD38+, n = 18) or vice versa (VLA-4+/CD38-, n = 13).

### Expression of VLA-4 but not CD38 defines high BM homing and human BM infiltration in discordant cases

**Figure 2:**
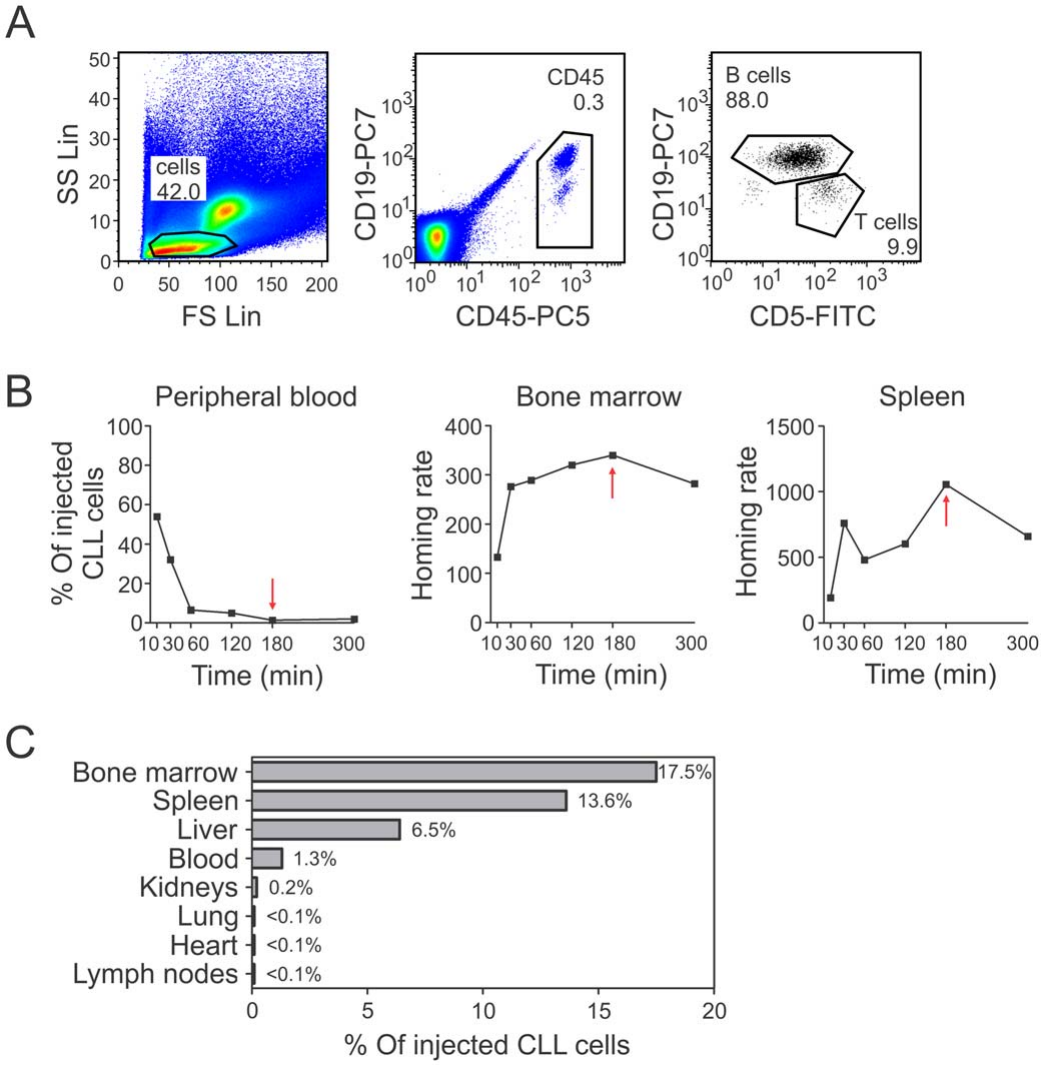
Homing capacity of CLL cells. (A) Representative plots of human CLL and T cells in a BM sample from a NOD/SCID mouse acquired by flow cytometry. Total mouse and human lymphocytes were gated in the FS/SS plot. Human cells were identified by anti-human CD45 staining and were analyzed for their CD19 and CD5 expression. CLL cells were defined as CD19+CD5+ and T cells as CD19-CD5+. (B) Time course experiment. Percent of injected human CLL cells detected in PB, and BM and spleen homing rates at the indicated time points. The red arrow marks lowest (PB) and highest (BM and spleen) cell numbers. (C) Percentage of injected CLL cells from a high-risk patient detected in various organs of a NOD/SCID mouse 180 min after injection. Homing rates were normalized as described [12]: number of human cells analyzed per 10^6^ mouse cells (total cells) per 10^6^ injected viable human target cells.

Having observed the phenotypic association of VLA-4 and CD38 in CLL cells, we investigated the individual as well as the combined contribution of these molecules to CLL cell dissemination. To this end we used *in vivo* short-term adoptive transfer assays of human CLL cells into immunodeficient mice, which we had previously established [12] to monitor the capacity of CLL cells to extravasate into BM and other organs that may provide an important niche for leukemic cells [14]. PBMCs of CLL patients were injected into the tail veins of NOD/SCID mice. Mice were sacrificed after the incubation period, and CLL cells were cytometrically identified in the murine organs by a combination of human-specific antibodies. A description of the gating strategy is depicted in Fig. 2A. First, we evaluated the kinetics of CLL homing using a patient sample with both high VLA-4 and CD38 expression. CLL cells rapidly left the blood and homed to BM and spleen within 3 hours (Fig. 2B). In all organs combined, we detected a total of 39.1% of the originally injected CLL cells of the sample. We found most cells in BM, spleen, and liver, little remained in the blood (Fig. 2C). Tiny fractions were located in the kidneys, lung, heart and lymph nodes (Fig. 2C), while no CLL cells could be detected in the peritoneal cavity, thymus or brain (data not shown).

**Figure 3:**
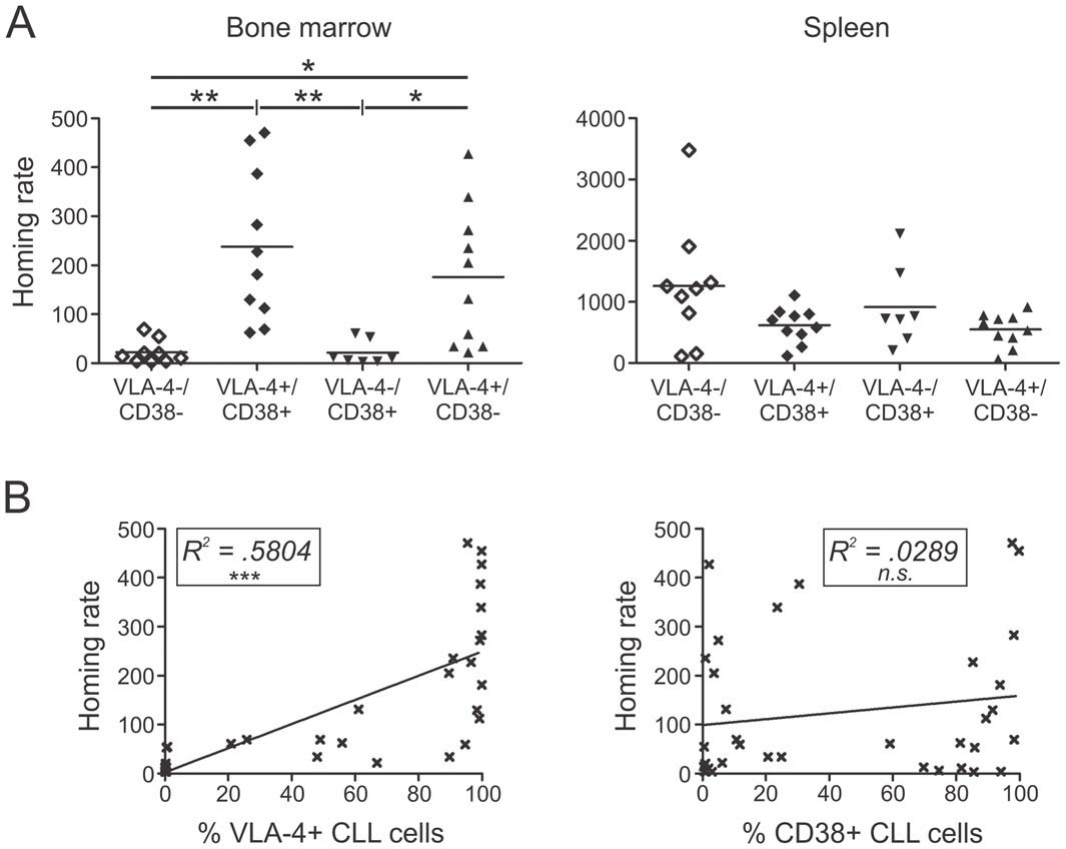
VLA-4, and not CD38, is essential for BM homing. Human CLL cells from different patients were injected into NOD/SCID mice. After 180 min mice were sacrificed and the number of human cells detected by flow cytometry. (A) BM and spleen homing rates of CLL cells from patients separated into four groups according to their VLA-4 and CD38 risk status (VLA-4-/CD38-, n = 9; VLA-4+/CD38+, n = 10; VLA-4-/CD38+, n = 7; VLA-4+/CD38-, n = 10; Anova, Kruskal-Wallis test, BM: p<.0001, Spleen: p = 0.1637, and Dunn's Multiple Comparison test). (B) Correlation of percent VLA-4+ CLL cells of patients used in homing assays to corresponding BM homing rates (n = 36, Linear regression, R^2^ = .5804, p<.0001) and correlation of percent CD38+ CLL cells of patients used in homing assays to corresponding BM homing rates (n = 36, Linear regression, R^2^ = .0289, p = .3208). Homing rates were normalized as described[12]: number of human cells analyzed per 10^6^ mouse cells (total cells) per 10^6^ injected viable human target cells. *, P<.05; **, P<.01; ***, P<.001.

Next, we compared the relative homing rates of different CLL patient samples after 3 hours with regard to their individual VLA-4 and CD38 expression. Although these parameters were associated in most patient samples, a significant proportion of patients showed a discordant VLA-4 and CD38 risk. In order to discriminate the roles of VLA-4 and CD38 in BM homing, we especially investigated the capacity of CLL cells from these discordant samples to home to BM. CLL cells from VLA-4-/CD38- patients showed significantly lower BM homing rates than those from VLA-4+/CD38+ patients (Fig. 3A). VLA-4-/CD38+ cells were hardly able to enter BM, while VLA-4+/CD38- samples displayed high BM homing rates, comparable to VLA-4+/CD38+ samples (Fig. 3A). In contrast, the spleen homing rates did not significantly differ between these four groups (Fig. 3A).

Of note, CLL cells from patients with combined high VLA-4 and CD38 risk had similar BM and spleen homing rates as healthy B cells (data not shown). We also verified these results by analyzing BM and spleen sections from several recipient mice by immunohistochemisty (Table 2).

**Table 2:** Detection of human B cells in organs of NOD/SCID mice via immunohistochemistry.

Human B cells (CD20, CD79a)	Bone marrow	Spleen
PBS control	−	−
CLL Low-risk	−	++
CLL High-risk	+	+
Healthy control	+	+

Exemplary analysis. - negative; + only single cells are positive; ++ more than one cell per high power field.

For further confirmation, we correlated the individual BM homing rates with the VLA-4 and CD38 expression levels. The higher the percentage of VLA-4 positive CLL cells, the more cells were able to home to the murine BM, while there was no significant correlation of BM homing and CD38 expression (Fig. 3B). In addition, pretreatment of human cells with the blocking anti-VLA-4 antibody, HP2.1, strongly antagonized BM homing (Fig. S1A), confirming our previous observation using a different antibody clone, HP1.2 [12]. Importantly, pertussis toxin, an inhibitor of Gαi protein subunit signal transduction which is usually coupled to chemokine signaling, almost completely abrogated CLL homing to the BM (Fig. S1B).

### Human BM lymphoid infiltration in VLA-4/CD38 discordant cases

**Figure 4:**
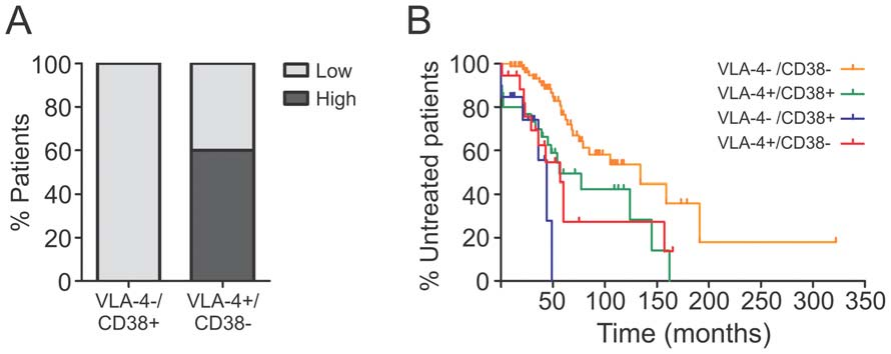
Human BM infiltration and treatment-free survival analysis of CLL patients with discordant VLA-4 and CD38 risk. (A) Human bone marrow biopsy histology was used to determine the BM lymphoid infiltrate rate of the individual VLA-4/CD38 discordant patients (n = 15). “High”: lymphoid infiltration of human BM greater than 50%, “Low”: ≤ 50%. (B) Kaplan-Meier analysis of treatment-free survival of CLL patients (n = 144) separated by their VLA-4 and CD38 risk (VLA-4-/CD38-, orange line, n = 83; VLA-4+/CD38+, green line, n = 30; VLA-4-/CD38+, blue line, n = 13; VLA-4+/CD38-, red line, n = 18; Logrank test for trend compared to VLA-4-CD38- control-group, p = .0004). When comparing the individual survival curves of all patient groups, all three groups with individual or combined high-risk parameters show significantly shorter treatment free-survival than patient group with VLA-4-/CD38- (VLA-4+/CD38+, p = .0042; VLA-4-/CD38+, p<.0001, VLA-4+/CD38-, p = .0026; Logrank (Mantel-Cox) test, adjusted significance level for multiple comparisions of survival curves by Bonferroni method  =  0.0083). There is no significant difference in treatment-free survival between the three high-risk groups (VLA-4+/CD38+ vs. VLA-4-/CD38+, p = .1861; VLA-4+/CD38+ vs. VLA-4+/CD38-, p = .9346; VLA-4-/CD38+ vs. VLA-4+/CD38-, p = .2350; Logrank (Mantel-Cox) test, adjusted significance level for multiple comparisions of survival curves by Bonferroni method  =  0.0083).

Our data suggest that in cases of discordant VLA-4/CD38 expression, only CLL cells with a VLA-4+ phenotype display an increased recirculation capacity to BM niches. We next wished to explore whether this characteristic clinically manifests in the extent of human BM infiltration with leukemic cells. Therefore, we used bone marrow biopsy histology from CLL patients to determine their BM lymphoid infiltrate rate. While we did not observe significant differences in the pattern of BM infiltration (categorized as diffuse, interstitial or nodular) between VLA-4-/CD38+ and VLA-4+/CD38- cases, we clearly detected a difference in the extent of lymphoid infiltrate in BM. As depicted in Fig. 4A, infiltration rates in VLA4-/CD38+ cases were all low (≤50%), whereas a lymphoid infiltration greater than 50% (“high” infiltration) could only be observed in the VLA-4+/CD38- cases (Chi-Square Test, p = .025). Notably, patients with VLA-4+/CD38+ expression as well as patients with discordant VLA-4 and CD38 expression displayed a significant shorter time to treatment than patients with VLA-4/CD38 low-risk but there was no significant difference between high-risk patient groups (combined or discordant) in terms of treatment-free survival (Fig. 4B).

### VLA-4 and CD38 expression is increased in BM compared to PB

**Figure 5:**
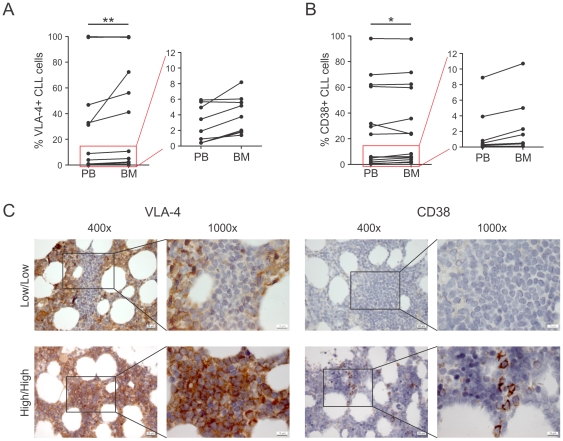
VLA-4 and CD38 are higher expressed in BM than in PB. Paired analysis of VLA-4 (A) and CD38 expression (B) on CD19+CD5+ CLL cells in PB and BM samples of patients by flow cytometry (n = 16, Wilcoxon signed rank test, VLA-4: p = .0069, CD38: p = .0362). Cases marked with red boxes in the left panels are drawn on a smaller scale in the right panels. (C) Immunohistochemical analysis of VLA-4 and CD38 expression in BM sections of a low-risk (VLA-4-/CD38-) CLL patient (top), and of a high-risk (VLA-4+/CD38+) patient (bottom) shown with indicated magnitudes. *, P<.05; **, P<.01.

The highly correlated VLA-4 and CD38 expression in CLL and the association of VLA-4 expression with leukemic BM infiltration prompted us to investigate whether there are differential compartment-specific alterations in VLA-4 and CD38 expression. We therefore analyzed the percentage of CD38+ or VLA-4+ cells in PB and BM aspirate-derived CLL cells of paired patient samples by flow cytometry. Both VLA-4 and CD38 expression were significantly increased in BM aspirates compared to the PB pool (Fig. 5AB), although this increase was small in most cases. Of note, remarkably increased VLA-4 expression was found in the BM of three samples with “intermediate” VLA-4 expression (Fig. 5A). As BM aspirates generally contain a relevant amount of PB that considerably dilutes the actual BM, we also performed immunohistochemistry on selected BM patient samples (Fig. 5C). In BM sections of a patient with very low VLA-4 and CD38 expression (<1.5%) and nodular CLL infiltrations, the VLA-4 negative CLL clone was clearly visible but was surrounded by high VLA-4 expressing BM areas. In contrast, the CLL clone and the surrounding BM areas were negative for CD38 in the same sample. In BM sections of a high-risk patient with diffuse infiltration, which is associated with accelerated disease[15], VLA-4 and CD38 expression in the CLL cells infiltrating the marrow could be confirmed by immunohistochemistry. VLA-4 was uniformly highly expressed, whereas CD38 expression was more diverse, i.e. in some areas CLL cells expressed high levels of CD38, whereas in other areas CD38 expression was largely negative.

### VLA-4 negative and CD38 positive subpopulations in BM show enriched Ki-67 expression

**Figure 6:**
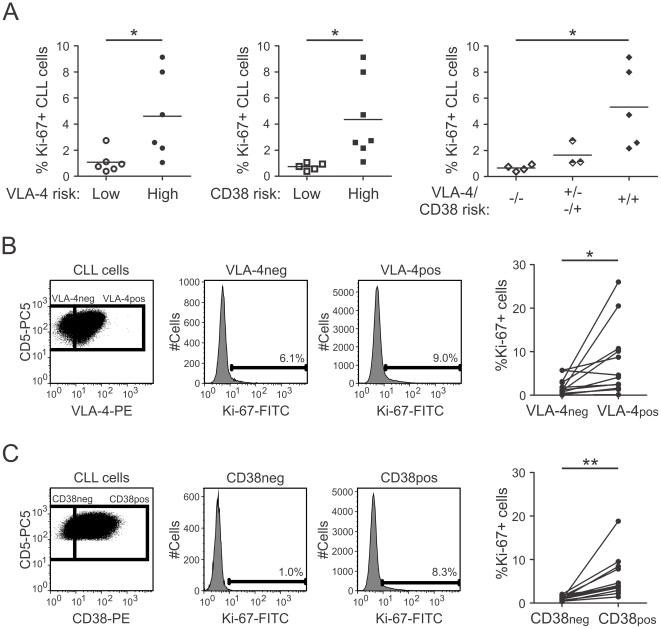
Ki-67 expression in BM aspirates is higher in CLL samples from high-risk patients, and VLA-4 negative and CD38 positive subclones express higher levels of Ki-67 in BM. MNCs from BM aspirates of CLL patients were stained with anti-CD19-PC7, CD5-PC5 and either VLA-4-PE or CD38-PE or respective isotype control-PE antibody and intracellulary stained with Ki-67-FITC or isotype control before flow cytometric analysis. (A) Left. Difference in Ki-67 expression in CLL samples from VLA-4 low (n = 6) and high-risk (n = 6, Unpaired t-test, p = .0298) patients as well as CD38 low (n = 5) and high-risk patients (n = 7, Unpaired t-test, p = .0278). Right. Difference in Ki-67 expression in CLL cells from patients with a combined low risk (-/-, VLA-4-/CD38-, n = 4), with discordant VLA-4 and CD38 risk (+/− −/+, n = 3), and VLA-4+/CD38+ (+/+, n = 5, ANOVA, One-way analysis of variance, p = .0233, Bonferroni's multiple comparision test). (B) Representative plot of CLL cells (CD19+/CD5+) analysed for their VLA-4 expression. VLA-4 negative and VLA-4 positive CLL cells were gated and further analyzed for their Ki-67 expression. (C) Representative plots for CD38 analysis. Percentage of Ki-67+ CLL cells in (A, right) VLA-4 negative and VLA-4 positive subclones within individual patients (n = 12, Paired t-test, p = .0275) or in (B, right) CD38 negative and CD38 positive subclones (n = 12, Paired t-test, p = .0048). neg, negative subclones; pos, positive subclones. *, P<.05; **, P<.01.

Since we found that both VLA-4 and CD38 were elevated in the BM, we also analyzed expression of the proliferation marker Ki-67 in CLL cells of BM aspirates by flow cytometry. Following the approach of Damle et al.[16], we tested both the Ki-67 expression dependent on the risk status and its expression within CLL clones of the individual samples. We first compared the % Ki-67+ CLL cells between the groups and found that Ki-67 expression in CLL cells (identified by their CD5, CD19 expression) was significantly higher in the VLA-4+CD38+ group than in the VLA-4-CD38- group (median Ki-67 expression 4.72% vs 0.65%) (Fig. 6A). Three patients with discordant VLA-4 and CD38 risk displayed intermediate Ki-67 expression (Fig. 6A).

Next, we determined Ki-67 expression in the VLA-4 negative and VLA-4 positive or CD38 negative and CD38 positive subfractions of individual CLL samples in BM aspirates by flow cytometry. The gating strategy, according to [16], is depicted in Fig. 6B, C. In the BM, Ki-67+ CLL cells were significantly enriched in VLA-4 positive (Fig. 6B), as well as in the CD38 positive, CLL subclones (Fig. 6C). While VLA-4 negative subclones had more diverse Ki-67 expression, CD38 negative subclones were nearly uniformly negative for Ki-67.

### High-risk CLL cells display higher spontaneous apoptosis rates in vitro

**Figure 7:**
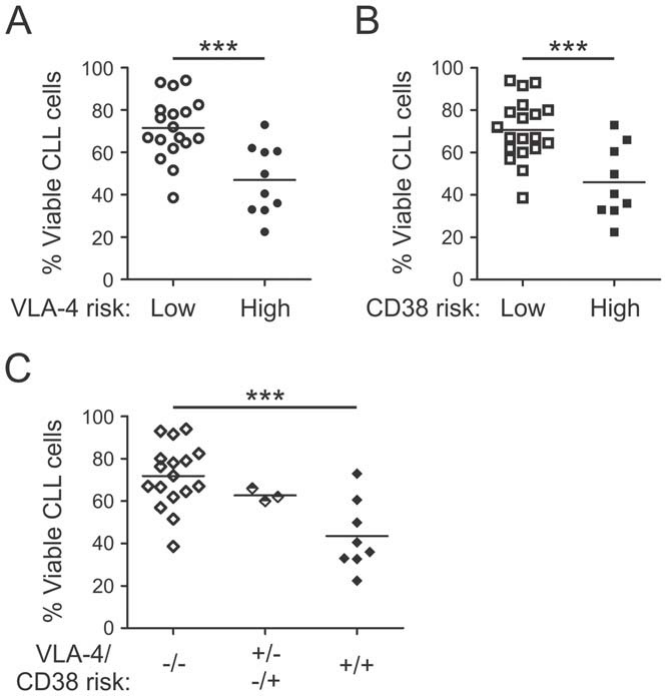
VLA-4 and CD38 high-risk CLL samples show higher spontaneous apoptosis rates. PBMCs from different CLL patients were cultured *in vitro* for 48 hours. Viability of CLL cells was analyzed by flow cytometry after 48 hours. Difference in viability of samples from (A) VLA-4 low (n = 18) and high-risk (n = 10; Unpaired t-test, p = .0004) patients and (B) and of CD38 low (n = 19) and high-risk (n = 9; Unpaired t-test, p = .0005) patient samples. (C) Difference in viabilty after 48 hours of cells from patients with a combined low risk (−/−, VLA-4-/CD38-, n = 17), with discordant VLA-4 and CD38 risk (+/−−/+, n = 3), and with combined high risk (+/+, VLA-4+/CD38+, n = 8, ANOVA, One-way analysis of variance, p = .0007, Bonferroni's multiple comparison test). ***, P<.001.

Our data suggest that expression of VLA-4 may allow circulating CLL cells to enter BM where they are likely to encounter anti-apoptotic and/or proliferative stimuli through interactions with accessory cells, thereby explaining the more aggressive clinical course of VLA-4+ patients. In this respect, co-culture with BM stromal cells has been shown to rescue CLL cells from otherwise rapid spontaneous apoptosis *in vitro* [17]. Consequently we were interested in the extent of stroma-derived survival benefits for the distinct risk groups. Therefore, we first compared the *in vitro* apoptosis rates of CLL cells from these risk groups. We observed no difference in the basal viability of CLL cells between high-risk and low-risk patients immediately following PBMC isolation (data not shown). However, after 48 hours in culture, the spontaneous apoptosis rates of CLL cells from high-risk patients were significantly higher than those of low-risk patients (Fig. 7). This was true for the VLA-4 (Fig. 7A) as well as CD38 high-risk groups (Fig. 7B). Cells from three patients with discordant VLA-4 and CD38 risk were tested and their spontaneous apoptosis rates were intermediate (Fig. 7C).

### Adhesion of CLL cells to stromal cells via VLA-4 confers only limited survival supports

**Figure 8:**
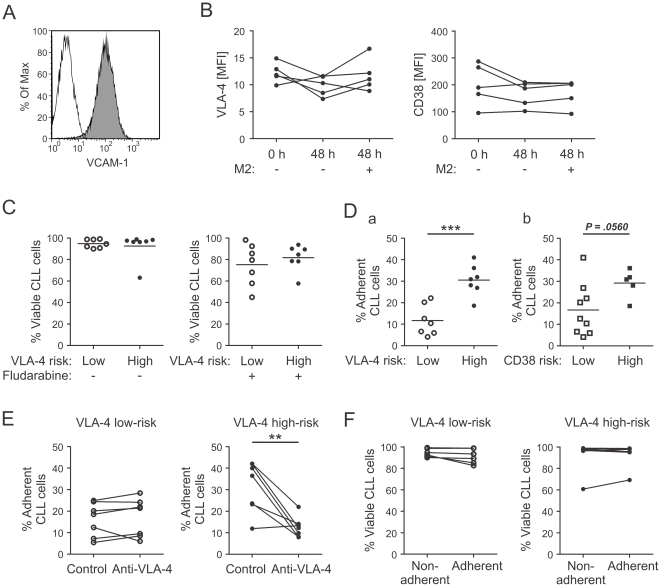
CLL cells from patients with different risk-groups are equally protected by stromal cells but cells from high-risk patients are able to adhere in higher numbers. (A) VCAM-1 expression on M2-10B4 cells was detected by flow cytometry. (B) PBMCs from CLL patients were cultured in the absence or presence of M2-10B4 cells for 48 hours. VLA-4 and CD38 expression levels on viable CD19+CD5+ CLL cells were determined by flow cytometry at indicated time points. (n = 5, Repeated Measures ANOVA, VLA-4, p = .0083; CD38, p = .1681; Bonferroni's Multiple Comparision Test) (C-F) PBMCs from CLL patients were co-cultured with M2-10B4 cells and after 48 hours viability and adhesion of CLL cells was determined by flow cytometry. (C) Viability of CLL cells from patients with low (n = 7) and high (n = 7) VLA-4-risk left untreated (Mann Whitney test, p = .9015) or treated with 5 µM fludarabine (Unpaired t-test, p = .4626). (D) Adhesion of CLL cells to M2-10B4 of different patient risk-groups (a, VLA-4, low, n = 7; high, n = 7; Unpaired t-test, p = .0003; b, CD38, low, n = 9; high, n = 5, Unpaired t-test, p = .0560). (E) Adhesion of CLL cells to M2-10B4 cells of VLA-4 low-risk (n = 7) or high-risk (n = 7) patient samples pretreated with isotype control (Control) or anti-VLA-4 antibodies. (Paired t-test, low-risk: p = ,4876; high-risk: p = .0059) (F) Viability of non-adherent and adherent CLL cells to M2-10B4 cells of VLA-4 low-risk (n = 7, Paired t test, p = .1186) or high-risk (n = 7, Wilcoxon signed rank test, p = .5781) patient samples. **, P<.01, ***, P<.001.

Next, we compared the protective effects of stromal cells on CLL cells from low- and high-risk patients. We cultured CLL cells together with the murine BM-derived cell line M2-10B4, a commonly used model system for tumor cell-marrow interactions [17], Expression of the VLA-4 ligand, VCAM-1, on the surface of M2-10B4 cells was confirmed by flow cytometry (Fig. 8A). We first wanted to investigate the influence of co-culture on the expression of VLA-4 and CD38, and the viability of CLL cells. In vitro culture of CLL cells alone for up to 48 hrs resulted in a slight but non-significant decrease in VLA-4 expression of the CLL cells (Fig. 8B). The additional presence of M2-10B4 cells did not significantly alter the VLA-4 expression. Likewise, CD38 expression was neither influenced by the culture time nor the presence of stromal cells (Fig. 8B). Next, we found that CLL cells from low- and high-risk groups were equally protected by stromal cells from spontaneous or fludarabine-induced apoptosis after 48 hours (VLA-4: Fig. 8C, CD38: data not shown). This implies, however, that the net effect of stromal cell protection was much greater in high-risk CLL cells, since high-risk CLL cells were significantly less viable than low-risk cells when cultured without stromal support (Fig. 7).

We found significantly increased adhesion of CLL cells from VLA-4 high-risk patients to M2-10B4 cells as compared to cells from low-risk patients (Fig. 8Da). The same was true for CD38 risk groups, but the difference was only borderline significant (p = 0.056, Fig. 8Db). The increased adhesion of CLL cells from VLA-4 high-risk patients was blocked when anti-VLA-4 antibodies were added, while adhesion levels of low-risk samples were unaffected (Fig. 8E). When we assayed the non-adherent and adherent fractions separately for cell viability, we found that CLL cells in both fractions where similarly protected by stromal cells against spontaneous and fludarabine-induced apoptosis (Fig. 8F and data not shown). Furthermore, the addition of VLA-4 blocking antibodies had no effect on CLL cell viability in the co-culture setting. Thus, VLA-4-mediated adhesion did not directly impact viability.

Collectively, our data suggest a prominent role of VLA-4, but not CD38, in the migration of CLL cells to supportive BM niches. However, VLA-4 mediated adhesion to BM stromal cells is not a dominant factor in protecting CLL cells from apoptosis *in vitro*.

## Discussion

Emerging evidence suggests that the proliferation and survival of CLL cells is dependent on their interplay with accessory cells and molecular factors in supportive niches.[18] It is therefore essential to define the molecular mechanisms underlying the trafficking of CLL cells into these niches. Among the plenitude of prognostic markers supposed to have a pathophysiological influence, VLA-4 and CD38 may be key players regulating tissue invasion and infiltration in CLL [19]. Since the majority of CLL samples show associated VLA-4 and CD38 expression, their individual roles in CLL cell migration and interactions with supportive accessory cells are still unclear. Therefore, in this study, we particularly analyzed samples with discordant VLA-4/CD38 expression. Collectively, our data argue for a higher propensity of high-risk CLL cells to infiltrate supportive BM niches as a result of their VLA-4 rather than CD38 expression, but only a limited role of VLA-4 in malignant cell-stroma interactions.

VLA-4 is a central player in B lymphopoiesis and is highly expressed in normal PB and BM B lymphocytes.[20] Thus, the variable VLA-4 expression and its applicability as a prognostic marker in CLL is quite a unique feature among B cell malignancies. In line with recent studies noting a high correlation between VLA-4 and CD38 expression,[2], [3] we detected not only a strong association between the risk groups but also a positive correlation of the individual expression levels (Fig. 1). Nevertheless, in about 20% of the cases we observed discordant VLA-4 and CD38 expression, and we specifically used these cases to explore the individual roles of these two molecules. We found that VLA-4 expression on its own was sufficient to allow entry into the BM, while CD38 expression was dispensable (Fig. 3A). In fact, the BM homing rate could directly be correlated with the extent of VLA-4 (Fig. 3B) but not CD38 expression and was completely abrogated by anti-VLA-4 antibodies and the Gαi inhibitor pertussis toxin (Fig. S1), suggesting that functional VLA-4 and Gαi-dependent chemokine signaling is needed for crossing the BM vasculature. The significant correlation between the extent of VLA-4 positivity of the sample and the BM homing capacity of the cells is in line with our previous observation of reduced circulation capacity of CLL cells at early Rai stages, which displayed lower VLA-4 expression than normal B lymphocytes.[12] More importantly, clinically, the VLA-4 state is directly manifested in the extent of human BM infiltration while the CD38 state did not influence it (Fig. 4A). Still, it is important to note that each prognostic marker on its own, VLA-4 or CD38, was sufficient to predict shortened time to treatment of the patients (Fig. 4B).

The exact role of CD38 in CLL pathophysiology remains an open question. In our setting, BM homing was not specifically blocked using anti-CD38 antibodies (OKT10, data not shown), which were previously shown to antagonize cell adhesion to hyaluronic acid and BM endothelium.[21] Yet, in a recent study, the homing of CLL samples to the BM could be abrogated with a high dose of a different anti-CD38 clone.[9] However, the authors did not analyze whether CD38 expression is needed for entry into the BM.[9] CD38 is a cyclic ADP-ribose that influences calcium signaling[6] and has the propensity to laterally associate with several molecules in membranal lipid rafts [22]. Our data clearly support the reported correlation between CD38 and proliferation [16], [23], which we observed to be stronger than that of VLA-4 and proliferation (Fig. 6). We therefore speculate that CD38 is primarily involved in calcium signaling during proliferation. Although CD38 may additionally act as an adaptor molecule that fine-tunes calcium signaling during chemokine-induced migratory responses [6], integrin-dependent signaling routes seem to be dominant and able to fully overrule its contribution.

Occasional *in vitro* chemoresistance of VLA-4 positive samples was observed in an earlier study [24]. In light of this study of de la Fuenta, our finding that VLA-4 high risk CLL cells are particularly sensitive to the absence of prosurvival stimuli from accessory cells (Fig. 7) was unexpected. However, our results are in complete consistency with the recent report by Coscia and colleagues who observed that high-risk CLL cells with an unmutated IGHV status were extremely vulnerable when removed from microenvironmental protection [25]. These differences between the risk groups might be based on alterations in microenvironment-induced NFkB signaling cascades [25]. Thus, disrupting microenvironmental interactions, potentially in combination with NFkB targeting, bears particular therapeutic potential for patients with a negative molecular prognostic signature. Despite higher adhesion rates of VLA-4 positive CLL cells to stromal cells, a VLA-4 dependent adhesion-mediated survival support could not be confirmed in our study (Fig. 8). Our results suggest a more complex scenario where CLL cells use VLA-4 for localization in protective niches rather than as a direct prosurvival molecule. This clearly does not reduce the therapeutic potential of VLA-4 antagonism, but rather suggests that the predominant effect of this interference will be reduction of malignant cell localization in protective microenvironmental niches such as bone marrow. We do also not exclude that VLA-4 mediated cell-cell contact may be a means to prime the stromal cells to secrete specific survival factors. VLA-4 low expressing cells appear to be less dependent on these cell-cell interactions and survival cascades.

In summary, our data suggest that VLA-4, rather than CD38, is mainly responsible for the recirculation of high-risk CLL cells into BM and for high BM infiltration observed in CLL patients. VLA-4 seems to be necessary to position those cells that are highly dependent on accessory survival signals at the appropriate supportive niche. Consequently, drugs that interfere with the homing properties of these cells, e.g., the anti-VLA-4 antibody Natalizumab[26], may be of particular benefit for this high-risk patient subgroup, especially in combination with current cytotoxic therapies. Moreover, Natalizumab could be used to target residual CLL cells surviving in the BM after conventional treatments[27], forcing them back into the blood stream where they become more vulnerable to treatment.

## Supporting Information

Figure S1 (A) BM homing rates of CLL cells from high-risk (VLA-4+/CD38+) patients (n = 3) that were either untreated or pretreated with anti-VLA-4 antibodies before injection into mice (Paired t-test, p = .0212). (B) BM homing rates of tumor cells from three high-risk (VLA-4+/CD38+) CLL patients that were either untreated or incubated with pertussis toxin (PTX) overnight before injection into mice (Paired t-test, p = .0227). Homing rates were normalized as described[12]: number of human cells analyzed per 10^6^ mouse cells (total cells) per 10^6^ injected viable human target cells. *, P<.05.
(TIF)

Table S1 **Detailed patient characteristics.**
(DOCX)

Methods S1 (DOC)
