# Supplementary figures and images for: Differential Bone Marrow Homing Capacity of VLA-4 and CD38 High Expressing Chronic Lymphocytic Leukemia Cells
Source: PLoS One. 2011 Aug 18;6(8):e23758. doi: 10.1371/journal.pone.0023758 (PMC3158106; doi:10.1371/journal.pone.0023758)

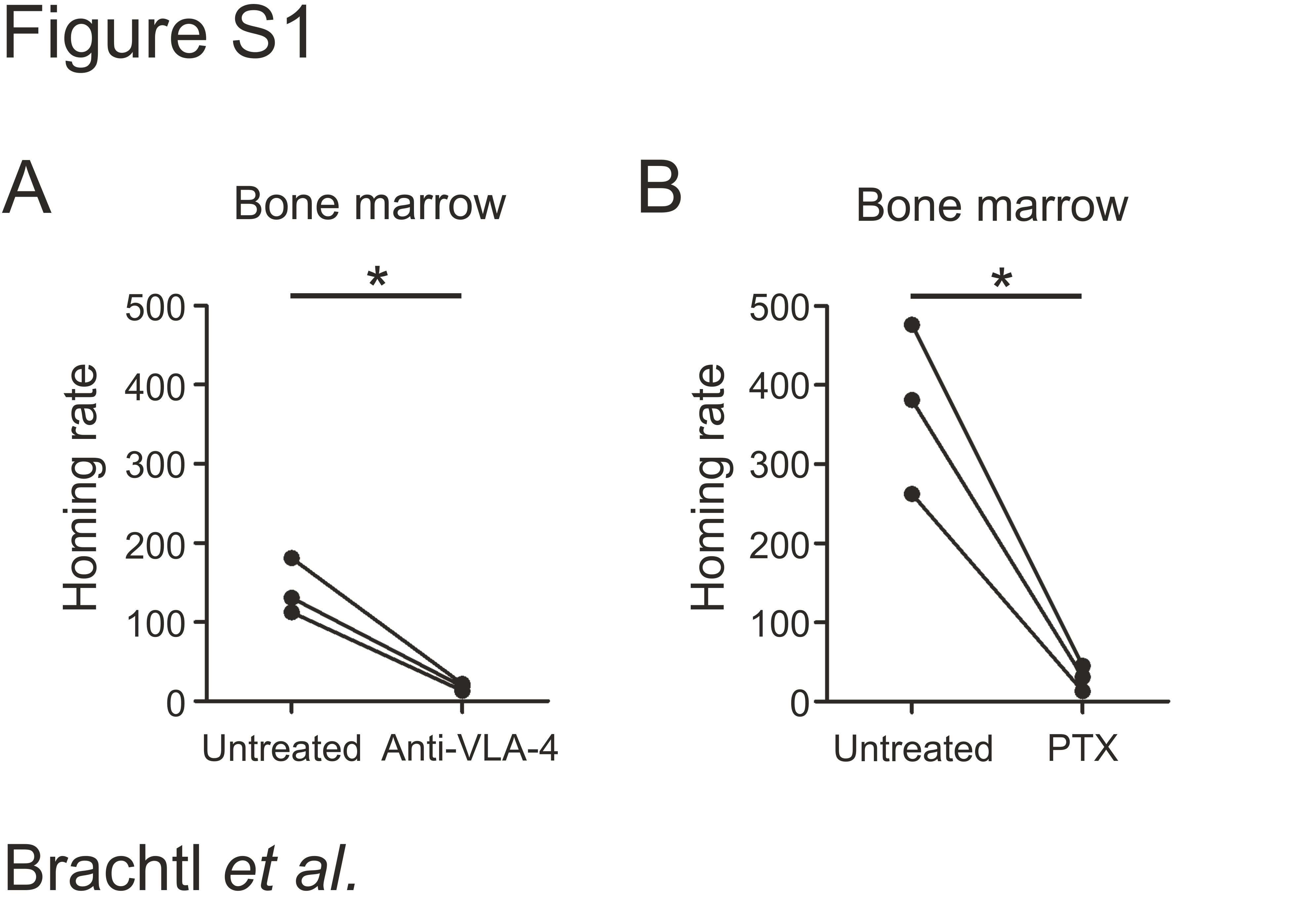

Supplement: Figure S1 — (A) BM homing rates of CLL cells from high-risk (VLA-4+/CD38+) patients (n = 3) that were either untreated or pretreated with anti-VLA-4 antibodies before injection into mice (Paired t-test, p = .0212). (B) BM homing rates of tumor cells from three high-risk (VLA-4+/CD38+) CLL patients that were either untreated or incubated with pertussis toxin (PTX) overnight before injection into mice (Paired t-test, p = .0227). Homing rates were normalized as described[12]: number of human cells analyzed per 106 mouse cells (total cells) per 106 injected viable human target cells. *, P<.05. (TIF) [file pone.0023758.s001.tif]
