# Supplementary material for: Differential Bone Marrow Homing Capacity of VLA-4 and CD38 High Expressing Chronic Lymphocytic Leukemia Cells
Source: PLoS One. 2011 Aug 18;6(8):e23758. doi: 10.1371/journal.pone.0023758 (PMC3158106; doi:10.1371/journal.pone.0023758)
Supplement: Table S1 — Detailed patient characteristics. (DOCX) [file pone.0023758.s002.docx]

Table S1. Detailed patient characteristics

| Pat.ID | Age | Sex | Chemonaive | Rai | IgVH | FISH | ZAP70 | VLA-4 | CD38 |
| --- | --- | --- | --- | --- | --- | --- | --- | --- | --- |
| Group 1: VLA-4 Low-risk/CD38 Low-risk | | | | | | | | | |
| 1 | 48 | F | No | 2 | MUT | absent | ND | Low | Low |
| 2 | 65 | F | Yes | 0 | MUT | absent | Low | Low | Low |
| 3 | 74 | M | Yes | 1 | MUT | absent | Low | Low | Low |
| 4 | 66 | F | Yes | 0 | MUT | del13q14 | ND | Low | Low |
| 5 | 78 | M | Yes | 1 | MUT | absent | ND | Low | Low |
| 6 | 61 | F | Yes | 1 | MUT | del13q14 | Low | Low | Low |
| 7 | 84 | F | Yes | 0 | MUT | absent | Low | Low | Low |
| 8 | 80 | M | No | 0 | MUT | absent | Low | Low | Low |
| 9 | 82 | F | No | 1 | MUT | absent | Low | Low | Low |
| 10 | 76 | M | No | 2 | MUT | del13q14 | Low | Low | Low |
| 11 | 64 | F | Yes | 1 | MUT | del13q14 | Low | Low | Low |
| 12 | 82 | F | Yes | 0 | MUT | del13q14 | ND | Low | Low |
| 13 | 86 | M | No | 0 | MUT | del13q14 | Low | Low | Low |
| 14 | 58 | M | Yes | 0 | MUT | del13q14 | Low | Low | Low |
| 15 | 64 | M | Yes | 2 | MUT | absent | ND | Low | Low |
| 16 | 80 | M | Yes | 2 | MUT | absent | Low | Low | Low |
| 17 | 60 | F | Yes | 1 | UMT | del13q14 | ND | Low | Low |
| 18 | 81 | M | No | 2 | MUT | del13q14 | Low | Low | Low |
| 19 | 71 | F | No | 2 | MUT | del13q14 | Low | Low | Low |
| 20 | 63 | M | No | 2 | MUT | del13q14 | Low | Low | Low |
| 21 | 77 | F | Yes | 2 | MUT | del13q14 | Low | Low | Low |
| 22 | 63 | M | No | 2 | UMT | del11q22, del13q14 | ND | Low | Low |
| 23 | 54 | M | No | 2 | UMT | absent | Low | Low | Low |
| 24 | 69 | F | Yes | 0 | ND | absent | Low | Low | Low |
| 25 | 72 | M | Yes | 2 | MUT | absent | Low | Low | Low |
| 26 | 70 | F | Yes | 0 | MUT | del13q14 | ND | Low | Low |
| 27 | 63 | F | Yes | 0 | MUT | absent | Low | Low | Low |
| 28 | 71 | M | Yes | 2 | MUT | del13q14 | Low | Low | Low |
| 29 | 75 | F | No | 3 | UMT | del13q14 | High | Low | Low |
| 30 | 86 | M | No | 1 | MUT | del13q14 | Low | Low | Low |
| 31 | 61 | M | Yes | 0 | MUT | del13q14 | High | Low | Low |
| 32 | 57 | M | Yes | 0 | MUT | del13q14 | Low | Low | Low |
| 33 | 75 | F | Yes | 0 | MUT | del13q14 | Low | Low | Low |
| 34 | 71 | M | Yes | 0 | MUT | absent | ND | Low | Low |
| 35 | 57 | M | No | 2 | MUT | del13q14 | Low | Low | Low |
| 36 | 58 | M | Yes | 0 | MUT | del13q14 | Low | Low | Low |
| 37 | 86 | M | Yes | 0 | MUT | del13q14 | Low | Low | Low |
| 38 | 71 | M | Yes | 0 | MUT | absent | High | Low | Low |
| 39 | 81 | M | No | 2 | UMT | absent | High | Low | Low |
| 40 | 69 | M | Yes | 1 | MUT | ND | ND | Low | Low |
| 41 | 69 | F | No | 2 | MUT | del17p, del13q14 | Low | Low | Low |
| 42 | 42 | M | Yes | 0 | ND | absent | High | Low | Low |
| 43 | 74 | M | Yes | 2 | ND | del13q14 | Low | Low | Low |
| 44 | 68 | M | Yes | 0 | MUT | del13q14 | Low | Low | Low |
| 45 | 69 | M | Yes | 1 | MUT | absent | Low | Low | Low |
| 46 | 70 | M | Yes | 2 | MUT | del17p, del13q14 | ND | Low | Low |
| 47 | 83 | F | No | 2 | MUT | del13q14 | Low | Low | Low |
| 48 | 80 | F | No | 1 | MUT | del13q14 | Low | Low | Low |
| 49 | 71 | M | No | 3 | ND | absent | Low | Low | Low |
| 50 | 60 | M | No | 1 | ND | del13q14 | Low | Low | Low |
| 51 | 60 | F | Yes | 0 | MUT | del13q14 | ND | Low | Low |
| 52 | 79 | F | Yes | 0 | ND | absent | High | Low | Low |
| 53 | 79 | F | Yes | 0 | MUT | absent | High | Low | Low |
| 54 | 46 | F | Yes | 0 | ND | del13q14 | High | Low | Low |
| 55 | 67 | F | No | 1 | UMT | del17p, del13q14 | Low | Low | Low |
| 56 | 84 | F | No | 0 | MUT | del13q14 | ND | Low | Low |
| 57 | 68 | M | Yes | 1 | MUT | absent | Low | Low | Low |
| 58 | 65 | M | Yes | 0 | MUT | del13q14 | Low | Low | Low |
| 59 | 46 | M | Yes | 0 | MUT | absent | High | Low | Low |
| 60 | 78 | F | Yes | 0 | MUT | absent | Low | Low | Low |
| 61 | 75 | F | Yes | 0 | MUT | ND | Low | Low | Low |
| 62 | 88 | F | No | 3 | MUT | del13q14 | Low | Low | Low |
| 63 | 61 | M | Yes | 0 | MUT | absent | Low | Low | Low |
| 64 | 83 | F | Yes | 0 | ND | absent | Low | Low | Low |
| 65 | 58 | M | Yes | 0 | MUT | absent | Low | Low | Low |
| 66 | 64 | M | Yes | 1 | MUT | del13q14 | High | Low | Low |
| 67 | 69 | F | Yes | 0 | MUT | absent | Low | Low | Low |
| 68 | 69 | M | No | 1 | ND | del11q22 | ND | Low | Low |
| 69 | 63 | F | Yes | 0 | ND | absent | Low | Low | Low |
| 70 | 85 | M | Yes | 0 | MUT | del13q14 | Low | Low | Low |
| 71 | 50 | F | Yes | 0 | MUT | del13q14 | High | Low | Low |
| 72 | 82 | M | Yes | 1 | MUT | del13q14 | Low | Low | Low |
| 73 | 54 | M | Yes | 1 | MUT | del13q14 | High | Low | Low |
| 74 | 81 | F | Yes | 2 | MUT | del13q14 | Low | Low | Low |
| 75 | 66 | F | Yes | 0 | MUT | del13q14 | Low | Low | Low |
| 76 | 87 | F | Yes | 2 | MUT | del13q14 | ND | Low | Low |
| 77 | 80 | M | Yes | 1 | MUT | del13q14 | Low | Low | Low |
| 78 | 71 | M | Yes | 0 | MUT | del13q14 | Low | Low | Low |
| 79 | 66 | F | No | 3 | UMT | ND | High | Low | Low |
| 80 | 79 | M | Yes | 0 | MUT | absent | Low | Low | Low |
| 81 | 71 | M | Yes | 1 | MUT | absent | Low | Low | Low |
| 82 | 73 | M | Yes | 0 | ND | del13q14 | High | Low | Low |
| 83 | 55 | F | No | 2 | MUT | del13q14 | Low | Low | Low |
| Group 2: VLA-4 High-risk/CD38 High-risk | | | | | | | | | |
| 84 | 69 | F | No | 2 | UMT | del11q22 | ND | High | High |
| 85 | 81 | F | No | 1 | UMT | tri12 | High | High | High |
| 86 | 57 | M | No | 2 | UMT | ND | High | High | High |
| 87 | 82 | F | Yes | 1 | MUT | absent | Low | High | High |
| 88 | 63 | M | No | 2 | UMT | del13q14 | High | High | High |
| 89 | 87 | F | No | 2 | UMT | absent | ND | High | High |
| 90 | 77 | F | No | 2 | MUT | ND | High | High | High |
| 91 | 73 | F | Yes | 1 | MUT | absent | Low | High | High |
| 92 | 79 | M | No | 2 | UMT | absent | ND | High | High |
| 93 | 85 | M | Yes | 2 | UMT | absent | High | High | High |
| 94 | 52 | M | No | 4 | UMT | del17p | ND | High | High |
| 95 | 83 | F | Yes | 0 | UMT | ND | ND | High | High |
| 96 | 74 | M | No | 2 | ND | absent | High | High | High |
| 97 | 68 | F | Yes | 0 | MUT | tri12 | Low | High | High |
| 98 | 73 | M | No | 2 | UMT | tri12 | High | High | High |
| 99 | 79 | F | Yes | 0 | UMT | tri12 | High | High | High |
| 100 | 62 | M | No | 4 | MUT | tri12, del13q14 | Low | High | High |
| 101 | 76 | M | No | 2 | UMT | del13q14 | High | High | High |
| 102 | 74 | M | Yes | 0 | MUT | del13q14 | High | High | High |
| 103 | 65 | M | Yes | 0 | MUT | absent | High | High | High |
| 104 | 65 | M | No | 2 | MUT | absent | High | High | High |
| 105 | 81 | M | Yes | 0 | MUT | absent | High | High | High |
| 106 | 75 | F | Yes | 0 | ND | del13q14 | High | High | High |
| 107 | 70 | F | Yes | 1 | MUT | absent | Low | High | High |
| 108 | 57 | M | No | 4 | UMT | absent | High | High | High |
| 109 | 69 | M | No | 4 | UMT | tri12 | High | High | High |
| 110 | 77 | F | No | 4 | UMT | del13q14 | ND | High | High |
| 111 | 67 | M | No | 4 | ND | del13q14 | High | High | High |
| 112 | 74 | F | No | 3 | UMT | ND | High | High | High |
| 113 | 60 | M | Yes | 1 | ND | absent | Low | High | High |
| Group 3: VLA-4 Low-risk/CD38 High-risk | | | | | | | | | |
| 114 | 49 | M | No | 2 | UMT | absent | ND | Low | High |
| 115 | 78 | M | No | 0 | UMT | absent | High | Low | High |
| 116 | 78 | F | Yes | 0 | UMT | absent | ND | Low | High |
| 117 | 80 | F | Yes | 0 | MUT | absent | Low | Low | High |
| 118 | 79 | M | Yes | 1 | MUT | del13q14 | Low | Low | High |
| 119 | 81 | M | No | 0 | ND | del11q22, del13q14 | Low | Low | High |
| 120 | 71 | M | No | 3 | ND | del13q14 | High | Low | High |
| 121 | 72 | M | No | 2 | UMT | del13q14 | High | Low | High |
| 122 | 64 | M | Yes | 0 | MUT | del13q14 | Low | Low | High |
| 123 | 60 | F | Yes | 0 | UMT | ND | High | Low | High |
| 124 | 68 | M | Yes | 1 | UMT | ND | ND | Low | High |
| 125 | 80 | F | No | 2 | UMT | del11q22, del13q14 | High | Low | High |
| 126 | 82 | M | Yes | 1 | ND | del11q22, del13q14 | High | Low | High |
| Group 4: VLA-4 High-risk/CD38 Low-risk | | | | | | | | | |
| 127 | 75 | M | No | 2 | ND | absent | ND | High | Low |
| 128 | 61 | M | No | 2 | MUT | tri12, del13q14 | ND | High | Low |
| 129 | 78 | F | Yes | 0 | ND | absent | Low | High | Low |
| 130 | 67 | M | Yes | 0 | MUT | del13q14 | Low | High | Low |
| 131 | 77 | M | Yes | 1 | UMT | absent | High | High | Low |
| 132 | 74 | M | No | 4 | MUT | del13q14 | Low | High | Low |
| 133 | 79 | M | No | 3 | UMT | del11q22 | ND | High | Low |
| 134 | 61 | M | Yes | 1 | MUT | absent | Low | High | Low |
| 135 | 58 | F | No | 2 | ND | tri12 | Low | High | Low |
| 136 | 81 | M | No | 1 | UMT | absent | High | High | Low |
| 137 | 81 | F | No | 2 | MUT | absent | Low | High | Low |
| 138 | 78 | F | No | 0 | UMT | del13q14 | Low | High | Low |
| 139 | 66 | F | Yes | 1 | UMT | ND | Low | High | Low |
| 140 | 84 | F | Yes | 1 | ND | tri12 | Low | High | Low |
| 141 | 83 | M | No | 3 | UMT | absent | High | High | Low |
| 142 | 72 | F | No | 4 | ND | del13q14 | High | High | Low |
| 143 | 73 | M | Yes | 0 | UMT | ND | High | High | Low |
| 144 | 68 | F | No | 1 | MUT | ND | High | High | Low |

M, *male*; F, *female*; MUT, *mutated IgVH*; UMT, *unmutated IgVH;* ND, *not determined;*
