# Supplementary material for: Differential Bone Marrow Homing Capacity of VLA-4 and CD38 High Expressing Chronic Lymphocytic Leukemia Cells
Source: PLoS One. 2011 Aug 18;6(8):e23758. doi: 10.1371/journal.pone.0023758 (PMC3158106; doi:10.1371/journal.pone.0023758)
Supplement: Methods S1 — (DOC) [file pone.0023758.s003.doc]

**Methods S1**

Mice – In vivo blocking experiments

For in vivo blocking experiments, PBMCs from CLL patients were preincubated for 30 minutes with or without 0.8 µg/mL blocking anti-VLA-4 (clone: HP2.1, Abcam, Cambridge, UK) or 100 ng/ml pertussis toxin (BIOTREND Chemikalien, Switzerland) overnight before cells were injected into the NOD/SCID mice as described. Control cells were incubated under identical conditions. Pre-treatments did not influence cell viability.

Patient data

Molecular risk parameters including cytogenetics, hypermutation status of immunoglobulin heavy chain genes (IgVH), and ZAP-70 expression were routinely analyzed in our laboratory. ZAP-70 risk was assessed as T cell /CLL expression ratio as previously recommended as consensus of the optimization of ZAP-70 evaluation procedures by multicenter efforts [1].

**Supporting Reference List**

1. Letestu R, Rawstron A, Ghia P, Villamor N, Leuven NB, et al. (2006) Evaluation of ZAP-70 expression by flow cytometry in chronic lymphocytic leukemia: A multicentric international harmonization process. Cytometry B Clin Cytom 70: 309-14.
